# From Mesenchymal Stromal Cells to Engineered Extracellular Vesicles: A New Therapeutic Paradigm

**DOI:** 10.3389/fcell.2021.705676

**Published:** 2021-07-20

**Authors:** Jancy Johnson, Mozhgan Shojaee, James Mitchell Crow, Ramin Khanabdali

**Affiliations:** ^1^Exopharm Ltd., Melbourne, VIC, Australia; ^2^Department of Biochemistry and Pharmacology, University of Melbourne, Parkville, VIC, Australia

**Keywords:** mesenchymal stromal cells, MSCs, extracellular vesicles, MSC-EVs, EV therapeutics, engineered EVs

## Abstract

Mesenchymal stromal cells (MSCs) are multipotent cells obtained from many tissues including bone marrow, adipose tissue, umbilical cord, amniotic fluid, and placenta. MSCs are the leading cell source for stem cell therapy due to their regenerative and immunomodulatory properties, their low risk of tumorigenesis and lack of ethical constraints. However, clinical applications of MSCs remain limited. MSC therapeutic development continues to pose challenges in terms of preparation, purity, consistency, efficiency, reproducibility, processing time and scalability. Additionally, there are issues with their poor engraftment and survival in sites of disease or damage that limit their capacity to directly replace damaged cells. A key recent development in MSC research, however, is the now widely accepted view that MSCs primarily exert therapeutic effects via paracrine factor secretion. One of the major paracrine effectors are extracellular vesicles (EVs). EVs represent a potential cell-free alternative to stem cell therapy but are also rapidly emerging as a novel therapeutic platform in their own right, particularly in the form of engineered EVs (EEVs) tailored to target a broad range of clinical indications. However, the development of EVs and EEVs for therapeutic application still faces a number of hurdles, including the establishment of a consistent, scalable cell source, and the development of robust GMP-compliant upstream and downstream manufacturing processes. In this review we will highlight the clinical challenges of MSC therapeutic development and discuss how EVs and EEVs can overcome the challenges faced in the clinical application of MSCs.

## Introduction

Mesenchymal stromal cells (MSCs) were identified more than 5 decades ago and have captured substantial attention among the scientific community since then due to their potential clinical applications (Friedenstein et al., 1970; Haynesworth et al., 1992). The ability of MSCs to differentiate into various cell types, and to support tissue function and repair, was quickly recognized. Since their initial discovery in bone marrow, MSCs from many other organs and tissues including adipose tissue, placenta, umbilical cord, Wharton’s Jelly, peripheral blood, skeletal muscle, skin, heart, liver, and the brain have also been isolated and studied (Baksh et al., 2004).

Mesenchymal stromal cells’ particular biological properties, including their self-renewal, proliferation and differentiation potential, make them attractive stem cell resources for pre-clinical and clinical studies that focus on the repair and/or regeneration of damaged tissue (Uccelli et al., 2008). The beneficial attributes of MSCs include their high resistance to oxidative stress, potent differentiation capacity, unique immunophenotypic characteristics, limited ethical constraints, low risk of tumor formation and powerful immunomodulatory activity (Trounson and McDonald, 2015). The medical interest in MSCs in indications ranging from cardiac conditions to neurodegenerative diseases has so far led to more than 1200 clinical trials being listed on clinicaltrials.gov.

The path from pre-clinical promise into clinical use has not been smooth, however. Multiple MSC therapeutics have failed to demonstrate efficacy in early or late clinical trials, in indications ranging from amyotrophic lateral sclerosis to stroke (Levy et al., 2020; FDA, 2021). Despite more than 30 years of work very few MSC therapeutic products have been approved for clinical use in any jurisdiction, indicating the significant pre-clinical and clinical challenges that remain to be resolved.

Despite the setbacks the field has experienced, the scale of effort still being applied to MSC-based therapeutics development is testament to the clinical promise that MSCs and their derivatives still hold. MSCs continue to show an excellent safety profile and remain the most studied stem cell population, particularly in the context of therapeutic use.

One of the most significant shifts in the MSC research field over the past decade has been the growing recognition that, rather than acting by engrafting and differentiating in sites of disease or injury to directly replace damaged cells, MSCs primarily exert their therapeutic effects via the release of paracrine factors (Yang et al., 2013). One of the major paracrine effectors are extracellular vesicles (EVs); nanoscale lipid-wrapped packages of lipids, proteins and nucleic acids that possess the same therapeutic properties as their parent cells (Bruno et al., 2009; Lai et al., 2010). As a cell-free MSC product, EV therapeutics pose few of the complications hampering the development of MSC cell therapies.

This review aims to highlight the ongoing issues associated with the development of MSC therapeutics and also to discuss how EVs isolated from MSCs overcome these issues to replace or complement cell-based therapies.

## Challenges in the Clinical Application of MSCs

Most MSC clinical studies have produced disappointing findings despite encouraging results in pre-clinical animal studies. Although 10 MSC therapeutics have received market approval in some jurisdictions (Table 1), in general low efficacy continues to blight MSC clinical trials. Some of the reasons proposed for this low efficacy are discussed below.

**TABLE 1:** List of regulator-approved MSC therapeutic products.

Drug name	MSC type	Indication	Country of approval (date)
Queencell	Autologous adipose MSC	Subcutaneous tissue defect	South Korea (March 2010)
Cellgram-AMI	Autologous bone marrow MSC	Acute myocardial infarction	South Korea (July 2011)
Cartistem	Allogeneic umbilical cord MSC	Knee articular cartilage defects	South Korea (January 2012)
Cupistem	Autologous adipose MSC	Crohn’s fistula	South Korea (January 2012)
Prochymal	Allogeneic bone marrow MSC	Graft-versus-host disease	Canada (May 2012); New Zealand (June 2012)
Neuronata-R	Autologous bone marrow MSC	Amytrophic lateral sclerosis	South Korea (July 2014)
Temcell HS Inj	Allogeneic bone marrow MSC	Graft-versus-host disease	Japan (September 2015)
Stempeucel	Allogeneic bone marrow MSC	Critical limb ischemia in Buerger’s Disease.	India (May 2016)
Alofisel	Allogeneic adipose MSC	Complex perianal fistulas in Crohn’s disease	Europe (March 2018)
Stemirac	Autologous bone marrow MSC	Spinal cord injury	Japan (December 2018)

### MSC Diversity

One factor that can lead to unexpected clinical outcomes is the significant heterogeneity that can exist between MSCs from different sources. MSCs isolated from different tissues show differences in their proliferative behavior and differentiation capacity in both *in vitro* and *in vivo* studies (Hass et al., 2011). Even when isolated from the same tissue type, significant differences in MSC populations have been observed between individual donors, with the characteristics of MSCs varying according to factors such as the donor’s age, health, sex, and body weight. For example, the age-associated deficits observed for MSCs include loss of key attributes such as proliferation and differentiation potential (Zhou et al., 2008). A study of aged bone marrow derived MSCs (BM-MSCs) recorded increased senescence, and a loss of bone formation capability (Stolzing et al., 2008). The decline of MSC function with age has significant implications for autologous use–particularly when considering that ill health itself can impair MSC function (van Rhijn-Brouwer et al., 2018).

Donor sex can also have an impact on the characteristics and function of harvested MSCs. In a rat model of lung inflammation, Female BM-MSCs reduced inflammation more effectively than BM-male MSCs (Sammour et al., 2016). A meta-analysis of human adipose tissue derived MSCs showed significant differences in the gene expression of cells from males and females, with the changes predicted to affect processes including inflammation, differentiation and cell communication (Bianconi et al., 2020).

### MSC Manufacturing Challenges

Once harvested, MSCs often need to be expanded to generate sufficient cells to be formulated into therapeutic doses. Treating a condition such as graft-versus-host disease may require tens of millions of cells per dose (Introna et al., 2014). Low cell harvest yield is particularly acute for BM-MSCs (Pittenger et al., 1999). Scale-up to a cell number sufficient for clinical use usually involves their proliferation in a large batch culture system. This process is lengthy and costly and therefore commercially unattractive. Additionally, MSC expansion and long-term culture to generate sufficient MSCs for clinical studies is often associated with increasing cell senescence and decreasing potency (Wagner et al., 2009).

Cost of MSC product manufacture and delivery is a significant barrier to its commercial viability. Depending on production scale and dose size, the cost of goods (COG) per dose varies dramatically, from US$485 to US$111,488 (Chilima et al., 2018). Technological advances such as bioreactors have been proposed to alleviate COG issues. This development may have the potential to improve MSC manufacturing output, and lower production costs (Chilima et al., 2018) but may not sufficiently address the COG issues. For example, hollow-fiber bioreactors were recently shown to be the least cost-effective manufacturing method due to high consumables and equipment costs with a COG almost double that required for a product to be commercially viable (Mizukami et al., 2018).

Culture medium development is another challenge for MSC production at the clinical level. Culture and expansion of MSCs has traditionally required media enriched with serum, but the complex and variable nature of this mixture of nutrients, growth factors and other constituents poses further challenges for maintaining product consistency. The development of serum-free media or chemically defined media is encouraging but they generally do not perform as well, especially for longer passages and scaling-up. They also put upward pressure on COG (Jung et al., 2012).

## Bioengineering to Boost the Clinical Potential of MSCs

The potential for clinical-scale MSC manufacture has been restricted by limited yield of donated cells, quality variation, and biosafety concerns regarding potential transmission of pathogens. An ideal cell source for industrial-scale MSC product manufacture should offer easy and unrestricted availability, have regulatorily acceptable provenance, present no biological safety risks, and be amenable to unlimited expansion while retaining its original “as harvested” phenotype. In a step toward this ideal, bioengineering approaches to increase both the yield and the homogeneity of MSCs are now being explored. For example, differentiation of induced pluripotent stem cells (iPSCs) to MSCs can be expanded to produce large quantities of cells thereby generating large quantities of highly homogenous MSCs (Ozay et al., 2019). Although concerns have been raised regarding the teratogenic potential of iPSC-derived cells, MSCs produced from iPSCs have been shown not to form teratomas or to show pro-tumor potential (Qingguo et al., 2015). After a series of pre-clinical studies, in indications including critical limb ischemia, asthma and organ transplant rejection, this approach was recently assessed in a Phase I clinical trial for acute steroid-resistant graft-versus-host disease (Bloor et al., 2020). No adverse events were reported during this study. In a related process, researchers have used a CRISPR/Cas9-based strategy to temporarily immortalize BM-MSCs, to readily expand these cells in long-term culture without the phenotypic changes that typically accumulate in high passage MSCs, before reversing the immortalization for potential therapeutic use (Hu et al., 2017). The safety of these reversibly immortalized cells now needs to be investigated before being used in clinical trials.

Despite the potential of engineered MSC sources to overcome specific limitations in MSC production, additional issues associated with MSC therapeutics persist. Immortalized live cell therapies may retain the issues associated with potential immunogenicity and tumorigenicity, and their engraftment qualities post-administration remain to be elucidated.

## EVs as a Novel Class of Therapeutic

Although it was previously believed that the therapeutic potential of MSCs was due to cell-to-cell contact or engraftment and differentiation of transplanted MSCs, it has become increasingly clear that MSC therapeutics’ prime mode of action is to release paracrine factors (Timmers et al., 2008; Caplan and Correa, 2011). In one non-human primate study of MSC infusion following total body irradiation, MSC engraftment levels were found to be below 3% (Devine et al., 2003). Similarly, in a mouse model of emphysema, Katsha et al. (2011) found that intratracheal injection with fluorescent labeled MSCs restored lung function, yet few engrafted MSCs and no differentiated cells could be detected in the lung beyond day 7 post-injection. The observed therapeutic effect was attributed to MSC paracrine factor release. Seminal papers reported that administration of conditioned media from cultured MSCs exerted the same beneficial effects as with whole cells (Gnecchi et al., 2006; Lai et al., 2010). Paracrine factors within the media were shown to be taken up by cells in the damaged tissue or by immune cells to promote cellular rejuvenation and restoration of tissue function. Prominent among the paracrine factors exerting these effects are EVs. Isolated MSC-EVs have shown to possess the same therapeutic potential as their parent cells (Bruno et al., 2009). This discovery has contributed to the interest in, and development of, EVs as medicines. As next generation, cell-free, MSC-based therapeutics, EVs have significant advantages in overcoming the limitations and risks associated with MSC-based cell therapy, as discussed below.

### EVs as a Cell-Free Application of MSCs

Extracellular vesicles are lipid bilayer-wrapped vesicles approximately 30–200 nanometers in size, released by virtually every cell type in the body. Once thought to be a mechanism for cellular refuse disposal, EVs are now known to be key mediators of cell-to-cell communication, delivering a cargo of lipids, proteins and nucleic acids that reflects their cell of origin. Although significantly simpler than live MSCs, EVs are still highly complex in composition and exert their effects by either transferring bioactive cargo such as functional protein and miRNAs that alter cell fate, or by modulating cell surface receptors and triggering intracellular signaling pathways (Xin et al., 2012; Guo et al., 2017).

**FIGURE 1:**
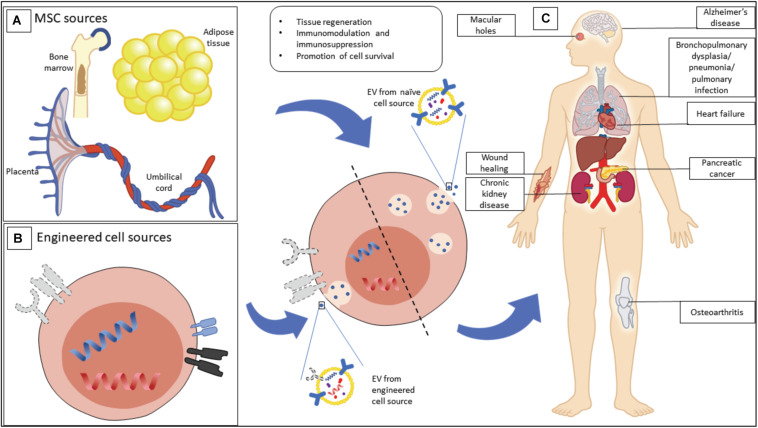
Mesenchymal stromal cell (MSC) EVs have therapeutic potential for a range of possible indications. **(A)** MSCs, as a source of MSC-EVs, can be isolated from a wide range of tissues, including bone marrow, adipose tissue, and birth-associated tissue. **(B)** MSCs can be engineered in order to enhance their output of EVs, or to produce EVs with particular properties. **(C)** Beneficial properties of EVs from naive and engineered sources are being explored for a range of clinical indications.

In the case of MSC-derived EVs, they have been found to share many attributes such as content, immunophenotype and unique homing ability (tropism) as their parental MSCs (Wei et al., 2021). As a result, their intrinsic therapeutic ability has been heavily investigated in recent years. MSC-EVs have been demonstrated as a potential treatment for several clinical indications including graft-versus-host disease (Kordelas et al., 2014), kidney injury (Nassar et al., 2016), myocardial infarction (Lai et al., 2010), systemic lupus erythematosus (Liu et al., 2015) and wound healing (Guo et al., 2017), and represent a novel therapeutic modality that could overcome roadblocks faced by MSC-based cell therapies (Figure 1).

#### The Advantages of EVs Compared to Cell-Based Therapies

The nature of EVs helps overcome several technological challenges faced by MSC-based therapy. Firstly, due to their phospholipid bilayer EVs are more resistant to damage caused by freeze-thaw cycles (Jeyaram and Jay, 2017) and possess high *in vivo* stability (Dang et al., 2020). In mice, intravenously injected MSC-EVs labeled with a bioluminescent dye were found to have an EV plasma half-life of 1.2–1.3 min due to their rapid uptake into tissues, where they were still detectable 24 h post-injection (Shelke et al., 2018). Secondly, as EVs do not self-replicate, they bypass the risk of endogenous tumor-formation that accompanies MSC-based therapy (Volarevic et al., 2018). Moreover, their small size as well as low or lack of expression of membrane histocompatibility complexes reduces the risk of inducing immune responses (Reis et al., 2016).

Extracellular vesicles also represent a novel strategy for hard-to-treat diseases for which there is a high unmet medical need. For example, in the treatment of neurological disorders some studies have shown that MSCs have difficulty transmigrating across the blood–brain barrier (BBB). In one study, MSCs were found to be retained in the lungs (Wang et al., 2010), while another reported that MSCs required a permeabilizing agent to cross the BBB (Cerri et al., 2015). Conversely, MSC-EVs have been shown to cross the BBB offering solutions in terms of route of administration and dosage (Moon et al., 2019). An example of their potential utility demonstrates that they can induce immunomodulatory and neuroprotective effects in a 3xTg model of Alzheimer’s disease (Losurdo et al., 2020) after systemic administration. MSC-EVs can also regulate neuronal cell apoptosis *in vitro* (Wei et al., 2020).

As EVs can be continually harvested from a MSC population, they can potentially overcome some of the issues of limited supply associated with MSCs themselves. As MSCs are highly amenable to modification, EV production can potentially be boosted to improve scalability. For example, the application of various stimuli to the parent cells, including physical stressors such as hypoxia or mechanical forces, or the addition of various small molecule modulators, can increase EV release (Zhu et al., 2017; Wang et al., 2020). MSCs have also been shown to produce significantly higher yields of EVs when grown in three-dimensional bioreactors rather than two-dimensional cell culture, and the EVs produced in these reactors can show enhanced therapeutic properties (Yan and Wu, 2020).

From the perspective of drug manufacturing, EVs offer several logistical advantages over cell-based therapies. These includes the possible addition of sterile filtration of the drug substance before aseptic filling, unfeasible for whole cells (Gimona et al., 2017). EVs are also suited to a wider range of storage conditions including flexibility in storage buffers as well as preservation techniques (Kusuma et al., 2018). Methods such as lyophilisation has been successfully utilized to store EVs (Frank et al., 2018) leading to an extended shelf-life and reduced costs due to a simplified cold chain (Kusuma et al., 2018). These characteristics suggest EV therapeutics could be developed as “off-the-shelf” products.

### Bioengineering to Broaden EV Therapeutic Utility

The therapeutic application of MSC-derived EVs is not limited to naïve, i.e., naturally secreted, EVs. The parent cell or its secreted EVs can be bioengineered to generate an EV product with enhanced or altered therapeutic properties. EV engineering strategies can be divided into two broad categories. Firstly, EVs can be engineered to alter their tropism and bias their uptake toward a target cell type. Alternatively, or in addition, EVs can be loaded with a particular therapeutic cargo.

#### Engineering EVs for Altered Tropism

A key factor determining the potential therapeutic utility of MSC-EVs is their fate once infused into the body. Several biodistribution studies have used dye- or bioluminescent-labeled naïve EVs to observe EV uptake by different tissues following infusion into animals (Wiklander et al., 2015; Shelke et al., 2018).

In a study on MSC-EVs labeled with a near-infrared lipophilic dye, more than 70% of EVs were found to accumulate in the liver when systemically injected into mice (Wiklander et al., 2015). Other animal studies have analyzed labeled naive EVs from a range of cell sources and shown that the EVs distribute widely through the body but accumulate primarily in the liver and spleen (Charoenviriyakul et al., 2017; Shelke et al., 2018). This natural tropism may be exploited to treat conditions associated with these organs. However, to enhance EV accumulation in other organs, EV engineering may be considered. This approach has been received favorably by some biopharma companies who are incorporating it into their developmental pipeline for “hard-to-treat disease,” e.g., cancer (Lewis et al., 2021).

Engineered EV (EEV) *in vivo* tropism may be altered by incorporating selected surface proteins onto their surface. One method for conferring targeting capabilities onto EVs is to manipulate the parent cell. One route is to create fusion proteins, in which the protein tail attaches or inserts into the EV membrane, and the head binds a receptor on the target cell type. In one early example, a fusion protein created between EV membrane protein Lamp2b and the rabies viral glycoprotein (RVG) peptide which binds the acetylcholine receptor on brain cells. These EEVs were used to target neurons, microglia and oligodendrocytes in the brain after systemic injection (Lydia et al., 2011). More recently, Lu et al. showed that a glycoprotein-g originally identified on the vesicular stomatitis virus, VSV-G, can be readily incorporated into nascent EV’s membrane by the parent cell. In addition, a large section of the VSV-G protein can be substituted for a “tropic” protein (Meyer et al., 2017). This approach has been shown to target EEVs to specific cell types including B cells, neurons and tumor cells (Yang et al., 2018).

Targeting proteins can also be incorporated onto EVs after their isolation from cell culture. Tian et al. (2021) recently showed that fusion proteins that bind the phosphatidylserine component of the EV lipid membrane can be used to target EVs to ischemic brain tissue after systemic injection, relieving inflammation. The targeting protein was attached to EVs by simple incubation. A similar approach has also been used to target tumor cells in a mouse model of glioblastoma (Zhilan et al., 2018).

As well as altering EV tropism, proteins can be added to the vesicle surface for a range of other applications. For example, EEVs with protein receptors on their surface have been developed as decoys to capture target molecules such as the pro-inflammatory cytokine IL-6, as a potential therapeutic for chronic inflammatory diseases (Conceição et al., 2021). Another study demonstrated that EEVs engineered to express CD47 on their surface have reduced systemic clearance, which extends circulation time to improve targeting of pancreatic cancer cells (Kamerkar et al., 2017).

#### Engineering EVs as Therapeutic Delivery Vehicles

Mesenchymal stromal cell-EVs are known to possess a cargo that can promote regeneration in damaged tissues, for example by lowering inflammation and inhibiting apoptosis to promote healing. EV engineering allows the possibility of loading EVs with a defined therapeutic cargo that could enhance this capability. MSCs have been engineered to overexpress microRNA-let7c, to generate EEVs which contain elevated levels of this microRNA. Infusion of these MSC-derived EEVs attenuated renal fibrosis in a mouse model of unilateral ureteral obstruction (Wang et al., 2016). More recently, MSC engineered to overexpress bone morphogenetic protein 2 produced EEVs with an enhanced capacity to promote bone regeneration (Chun-Chieh et al., 2020).

However, EV drug loading methodologies also enable the potential development of EEV therapeutics for a wide range of indications beyond tissue regeneration and healing. The key characteristics of EVs–non-immunogenic capsules able to deliver cargo to a target cell type–appear to make them ideal as drug delivery vehicles, such as for targeting tumor cells.

Many therapeutics, from small molecule drugs to RNAs, need to be shepherded to their site of action, either to overcome unfavorable pharmacokinetics, as protection from metabolic breakdown, or to reduce off-target effects. Synthetic delivery systems such as liposomes or nanoparticles have been limited by poor stability in storage, toxicity, poor efficacy and a limited capability to deliver cargo to target tissues other than the liver (Setten et al., 2019).

As knowledge surrounding EV biogenesis and mechanisms of cargo sorting has increased, new strategies have emerged to generate EEVs that contain a defined therapeutic cargo. The techniques associated with the generation of EEVs include incubation with drugs for delivery (Pascucci et al., 2014) or transfection of the parent cells to allow specific small RNA and small molecules to be incorporated into their EVs. For example, MSCs have been engineered to produce EVs enriched in the microRNA miR-379, a potent tumor suppressor. In a mouse model of breast cancer, systemic administration of the miR-379 EEVs significantly reduced tumor growth–whereas administration of the engineered parental MSCs themselves had no impact on tumor growth (O’Brien et al., 2018).

In head-to-head testing, EVs were recently shown to be superior to liposomes for RNA transfer, delivering a cargo of RNA several orders of magnitude more efficiently into cells (Murphy et al., 2021). The biggest hurdle, the researchers noted, was to efficiently load the RNA cargo into EVs–but emerging technologies can offer highly effective ways to accomplish EV RNA loading. Nguyen and Ferguson at the University at Buffalo identified RNA sequences that are specifically loaded by cells into EVs. By attaching therapeutic RNA to these sequences, up to 100-fold gains in loading of the therapeutic RNA into EVs was achieved (Nguyen and Ferguson, 2018).

Extracellular vesicle loading can also take place after EV isolation from cell culture. Techniques including freeze-thawing, sonication, electroporation, osmotic shock and saponin permeabilization have been developed to temporarily disrupt the EV membrane sufficient for uptake of a therapeutic cargo. However, as the cargo loading method itself may alter the biophysical characteristics or biological function of treated EVs, and the loading efficiency can vary between different types of cargo, care must be taken to determine the appropriate cargo-loading methodology in each case (Franziska et al., 2021).

### Clinical Application of EVs and EEVs

The progress made in EV pre-clinical studies and the additional advantages of EVs relative to whole cell therapeutics has led to a growing number of MSC-EV human clinical trials. Over 80 studies have been registered at the www.ClinicalTrials.gov database to assess the therapeutic effects of EVs in several therapeutic arenas. MSC-EVs have demonstrated acceptable safety and tolerability profiles, and promising signs of therapeutic efficacy (Table 2).

**TABLE 2:** List of registered clinical trials using MSC derived EVs.

EV source	Application	Clinical phase	Status	References
BM-MSC	Graft-versus-host disease	Individual patient	Completed	10.1038/leu.2014.41
Wharton’s jelly MSC	Chronic ulcer wounds	Phase I	Completed	NCT04134676
AD-MSC	COVID-19 associated pneumonia	Phase I	Completed	NCT04276987
MSC	COVID-19 associated pneumonia	Phase I/II	Completed	NCT04491240
UC-MSC	Chronic kidney disease	Phase II/III	Completed	10.1186/s40824-016-0068-0
UC-MSC	Macular holes	Early Phase I	Ongoing	NCT03437759
AD-MSC	Periodontitis	Early Phase I	Ongoing	NCT04270006
MSC	Pancreatic cancer	Phase I	Ongoing	NCT03608631
BM-MSC	Tolerance study on aerosol inhalation of MSC-EVs in healthy volunteers	Phase I	Ongoing	NCT04313647
MSC	Cerebrovascular disorders	Phase I/II	Ongoing	NCT03384433
AD-MSC	Pulmonary infection	Phase I/II	Ongoing	NCT04544215
AD-MSC	Alzheimer’s disease	Phase I/II	Ongoing	NCT04388982
UC-MSC	Dry eye in patients with chronic graft-versus-host disease	Phase II	Ongoing	NCT04213248
BM-MSC	Bronchopulmonary dysplasia	Phase I	Active, not recruiting	NCT03857841
BM-MSC	COVID-19 associated acute respiratory distress syndrome	Expanded access protocol	Available	NCT04657458
UC-MSC	Type I diabetes	Phase II/III	Unknown	NCT02138331
AD-MSC	Osteoarthritis	n/a	Not yet recruiting	NCT04223622
MSC	Multiple organ dysfunction syndrome following aortic dissection repair	n/a	Not yet recruiting	NCT04356300
AD-MSC	Osteoarthritis	Phase I	Not yet recruiting	NCT04223622
BM-MSC	Dystrophic epidermolysis bullosa	Phase I/II	Not yet recruiting	NCT04173650
BM-MSC	COVID-19 associated acute respiratory distress syndrome	Phase II	Not yet recruiting	NCT04493242

In one early study, for example, repeated injections of MSC-EVs demonstrated significant improvement in a patient with severe therapy-refractory acute graft-versus-host disease. The patient’s pro-inflammatory cytokine levels and disease symptoms improved markedly after MSC-EVs therapy, and the patient’s improvement remained stable for at least 4 months after treatment (Kordelas et al., 2014). In a Phase II/III placebo-controlled clinical trial, umbilical cord blood derived MSC-EVs administration was shown to be safe, and to modulate inflammation and improve kidney function, in patients with chronic kidney disease (Nassar et al., 2016).

Studies currently underway include a safety study of the use of bone marrow MSC-EVs to treat bronchopulmonary dysplasia, a chronic lung disease that mainly affects premature babies (NCT03857841); a Phase I study of MSC-EVs loaded with a siRNA therapeutic for patients with pancreatic cancer (NCT03608631); and a study to assess MSC-EV administration for improvement of disability in patients with acute ischemic stroke (NCT03384433).

There are also studies underway to test inhaled MSC-EVs in patients with acute respiratory distress syndrome (ARDS) associated with severe cases of COVID-19 (NCT04602442; NCT04602104). Table 2 summarizes clinical trials that have been performed using EVs isolated from MSCs.

Extracellular vesicles from other sources than MSCs have also been used in clinical trials. Significantly, in addition to the early phase trials conducted by academic groups, several trials of experimental EV therapeutic products developed by biopharmaceutical companies are now underway, reflecting the increasing maturity of the therapeutic EV field. These trials include platelet derived EVs tested by Exopharm in two Phase I clinical trials for wound healing applications (ANZCTR, 2019, 2020). Codiak Biosciences is currently investigating engineering the HEK293 cell line for their clinical applications, and in late 2020 commenced two Phase I clinical trials testing the safety and efficacy of EEVs for certain cancers (Clinicaltrials.gov, 2020).

Despite rapid development in the EV field and the commencement of clinical trials, several challenges remain and should be resolved before EVs can achieve widespread clinical utility. These challenges are discussed in the following section.

## Challenges in the Clinical Application of EVs

Although regulatory approval and safety of clinical application of EVs appears feasible, and may be simpler than cell-based therapy, important barriers that need to be addressed for clinical GMP-grade production of EVs include selection of the starting cellular material and optimizing cell culture, purification, quantification, and quality control (Gowen et al., 2020). Understanding and determining the quantity, purity and potency of EVs, by the development of appropriate assays, will be an important part of the quality control criteria (Gimona et al., 2021). Further challenges include issues of EV preservation and long-term stability, as we also go on to describe below.

### Identifying Ideal EV Cell Sources

As EV are a secreted product of cells, their manufacture is heavily dependent on the ability to produce large quantities of cells in ways that do not alter their phenotype. However, the opportunities for producing large quantities of stem-cell based conditioned medium with which to undertake meaningful scale-up of EV production are limited.

Generally, for stem cell derived EVs, the potential cell sources for EV production are either MSCs, or pluripotent stem cells (PSCs) such as embryonic stem cells (ESCs) or iPSCs derived lineages. In each case there are advantages and disadvantages associated with their clinical use, which are summarized in Table 3.

**TABLE 3:** Comparison of cell sources for large scale production of EVs.

	Somatic tissue derived MSCs	ESCs	iPSCs	ESC/iPSC derived MSCs	HEK293
**Quantity**	Variable, depending on donors	Reliable, can be derived from a single hPSCs line	Reliable, can be derived from a single hPSCs line	Reliable, can be derived from a single hPSCs line	Reliable, can be derived from a single hPSCs line
**Pathogens**	Possible and hard to control from sources	Rare and easy to control from sources	Rare and easy to control from sources	Rare and easy to control from sources	Rare and easy to control from the sources
**Cell number**	Limited	Unlimited	Unlimited	Unlimited	Unlimited
**Cell Homogeneity**	Medium	Medium	Medium	High	High
**Cost**	High	Medium	Medium	Medium	Low
**Differentiation efficiency**	High	High	High	Low	Low
**Proliferation**	Slow	Fast	Fast	Fast	Fast
**Immunomodulatory effects**	High	Low	Low	High	?
**Potency**	Medium	High	High	High	?
**Senescence**	Faster	Slow	Slow	Slow	Slow
**Genome editing**	Hard	Easy	Easy	Easy	Easy
**Level of risk overall**	Low	High	High	Medium	Medium
**References**	Mastrolia et al., 2019	Thomson et al., 1998	Keisuke et al., 2007; Akira et al., 2013	Xing-Liang et al., 2018; Ozay et al., 2019; Bloor et al., 2020	Jing et al., 2016; Magdalena et al., 2020

Primary or somatic MSCs may not be an ideal cell source for EV manufacture at clinical scale due to their limited lifespan, heterogeneity and batch-to-batch or donor-to-donor variations. One approach to increase the yield of EVs from MSCs is to immortalize MSCs using the hTERT method or a CRISPR/Cas9-based strategy, to enable long term cell culture and scaled up EV production. Recently, EVs from immortalized MSCs were shown to be non-tumorigenic both *in vitro* and *in vivo*, neither promoting nor inhibiting tumor growth (Tan et al., 2021).

Alternatively, pluripotent cells could be exploited as source cells. As stated previously, the ideal cell source for industrial-scale EV production should have unrestricted availability, regulatorily acceptable provenance, present no biological safety risks, and be amenable to unlimited expansion while retaining its original “as harvested” phenotype. The only cell types which can have most or least some of these characteristics are ESCs and iPSCs. The main advantage of these cells is their unlimited capacity and growth. The main disadvantages of these cells are the ethical concern in the case of ESCs, and the likelihood of immune responses and risk of teratoma for iPSCs.

An approach to exploit the benefits of pluripotent cells, but navigate their disadvantages, could be to incorporate iPSCs as a source of MSCs for EV manufacture. This approach might increase the yield and the homogeneity of the cell source for industrial scale production of EVs. The same scenario can also be applied to ESCs, by directly differentiating ESCs to MSCs. The MSCs derived from these sources have shown similar properties to adult MSCs, including their differentiation potential and immunomodulatory effect (Bloor et al., 2020). Several studies have demonstrated that these cell lines have superior efficacy over MSCs derived from somatic tissues (Jiang et al., 2019). The unique advantages of PSC derived MSCs over adult MSCs include unlimited supply, high purity, lower cost and most importantly, scalable production. The process of manufacturing clinical GMP-grade MSCs involves donor identification, screening, tissue harvest, cell isolation, purification and expansion which require a theater and clinician involvement as well as GMP facilities each time. Since this whole process must be repeated several times due to the limited expansion of adult MSCs compared with iPSC derived MSCs, this can increase the cost of GMP-grade production of adult MSCs compared with iPSC derived MSC line. These factors are all important for the development of a cell system for MSC-EV manufacture.

#### Alternate EV Cell Sources for EEV Applications

For therapeutic applications in which EVs are employed for their drug delivery capabilities rather than for their inherent regenerative properties, a broader range of EV cell sources can be considered. One potentially important cell source being explored for the mass production of EEVs are human stable cell lines such as HEK293. Currently, HEK293 cells are predominantly used for recombinant protein production and vector transfection. HEK293 cell derived EVs are a promising platform from which to produce EEVs modified specifically for the clinical indication of interest.

The main advantages of HEK293 are that this cell line proliferates rapidly, can be easily grown in serum-free suspension culture, and importantly is FDA-approved as a human cell line for recombinant protein production. As such, this cell type has become a particular focus for biotechnology companies developing EEV therapeutics. For instance, Codiak Bioscience is using HEK293 to produce engineered EVs as a potential cancer therapy and is conducting clinical trials using HEK293 engineered cells as an EEV source for the treatment of cutaneous T cell lymphoma and solid tumors (Dooley et al., 2021). In addition, ILIAS Biologics researchers and their collaborators have used HEK239-derived EEVs as a platform for several experimental therapeutics. These EEVs were recently used to deliver an inhibitor of transcription factor NF-κB, to prolong pregnancy in a mouse model of pre-term birth (Sheller-Miller et al., 2021).

Although the HEK293-derived EEV production system may offer potential manufacturing advantages, the potency, efficacy and associated biological risk factor of EV generated from this cell line need to be evaluated in both *in vitro* and *in vivo* systems.

### Meeting GMP-Grade Processing Requirements for Clinical EV Production

Once a reliable cell source for industrial-scale EV production is identified, issues remain in the upstream and downstream processing and quality control of the resulting EVs.

Purity is one significant issue that needs to be addressed for GMP-grade production of EVs. Research labs engaged in EV research, development and pre-clinical studies predominantly use media formulations containing fetal bovine serum (FBS) for cell culture and EV collection, which cannot be applied for clinical application (Gottipamula et al., 2013; Pachler et al., 2017). Although ultracentrifuged, EV-depleted FBS is often used by researchers and academician, not all the EV contaminants can be removed (Zhiyun et al., 2016; Shelke et al., 2018). The use of human platelet lysate (hPL) instead of FBS may solve the problem (Torreggiani et al., 2014). Ideally when manufacturing GMP-grade EVs, serum-free and chemically defined media are safest for EV collection, preventing the inadvertent introduction of contaminating EVs in the source serum (Pachler et al., 2017). However, switching from serum to serum-free cell culture media causes cell stress which in turn may lead to production of EVs with different cargo and profile. Therefore, cell culture conditions must be carefully considered. Large-scale culture systems that align with GMP requirements for the production of master and working cell banks also need to be adopted. Hollow fiber and stirred-tank bioreactors are the more promising approaches because they are closed, scalable, GMP-compatible systems that provide a high surface-to-volume ratio for cell growth (Mendt et al., 2018).

Large-scale isolation and purification of EVs is one of the most significant challenges in downstream processing for clinical GMP-grade EV production. Ultracentrifugation (UC) remains the most popular method of EV isolation, despite ongoing concerns over product purity, and that UC protocols are not directly scalable for clinical-scale manufacture (Casulli et al., 2018; Colao et al., 2018). Alternative purification methods such as those based on scalable and high throughput ion exchange protocols and size exclusion chromatography with membrane filtration such as tangential flow filtration (TFF), are being investigated as a promising way around this significant roadblock (Law et al., 2021). EV purification should ideally utilize methods such as filtration and chromatography that are currently available for the manufacture of biologics, as these methods are already demonstrated to be GMP-compliant.

As different isolation and purification methods have an impact on EV characteristics and produce different population of EVs, the need for standardized GMP is emphasized (Agnes et al., 2018). MISEV guidelines lay out basic standards for EV markers, but specific standardized universal markers are still lacking for EV characterization, and further investigation is required to fully characterize EV populations to establish their identity.

Quantification of EVs is another fundamental issue which remains challenging. How to accurately measure and assess EV purity is a critical issue when evaluating EV dosage for both pre-clinical and clinical applications. At present, there is no single method that can accurately measure EVs. One of the most common approaches to measuring EVs is determining the total amount of proteins and particle numbers. There are several methods and instruments measuring EV numbers including nanoparticle tracking analysis (NTA), resistive pulse sensing (RPS), and dynamic light scattering (DLS). However, copurification of other proteins by measuring total protein concentration and current particle tracking approaches might be biased toward a designated EV size range which does not discriminate EVs from other nanoparticulate materials. Recent developments in this field include nanoflow cytometry, imaging flow cytometry, and ExoCounter with optic disk technology to quantify EVs (Sotiris et al., 2018; Hartjes et al., 2019). A combination of flow cytometry-based methods for analyzing the membrane surface markers and quantification of EVs may represent improvements in the assessment of EV purity and yield.

For GMP compliance, fill and finish of the final product should be performed in a closed system. This might present as a logistical challenge for small biotech companies and academic labs operating at research scale (Rohde et al., 2019). Overall, the currently available methods and technologies for purification, quantification and characterization of EVs are inconsistent and GMP standards need to be developed that are reproducible, practical and scalable for clinical GMP-grade EV application. However, with better know-how and strategic decisions early on in development, GMP-grade EV preparations can be successfully produced for clinical administration (Mendt et al., 2018).

Finally, storage and long-term stability of EVs is another important issue if EVs are to be used as a third party “off the shelf” product. EV quantity decreased in a dose-dependent manner at room temperature, at 4°C, −20°C, but not when frozen in −80°C, therefore most protocols store EVs at −80°C long term without any reported changes in EV profile and structure (Lõrincz et al., 2014). Preservation condition and storage solution buffer is also very important as EVs are sensitive to shifts in pH. The majority of published studies have used phosphate-buffered saline (PBS) and some have used sucrose and trehalose buffers (Steffi et al., 2016; Busatto et al., 2018; Li et al., 2019). Preservation conditions need to be further optimized and validated in both *in vitro* and *in vivo* assays to ensure post-thaw potency of the EVs product is retained (Chung et al., 2021). Alternatively, EV lyophilization has been successfully utilized to store EVs and may offer a simpler option for transport and storage (Frank et al., 2018; Kusuma et al., 2018). Standardized GMP-grade methods to define specific storage condition including temperature, buffer solution, pH and duration must be addressed for clinical applications of EVs to be advanced.

## Conclusion

Experimental MSC therapies continue to show promise in pre-clinical research. This promise, however, is still to translate into widespread clinical use. The inherent complexity of a live cell product presents considerable challenges to successful therapeutic translation. Although cell engineering approaches may address some of these challenges, the potential risks associated with cell-based therapeutics, such as tumorigenicity and undesired differentiation, and limitations such as rejection of cells and poor engraftment, will remain.

Mesenchymal stromal cell-EVs, as a cell-free product, offer a potential pathway through these challenges. An increasing number of studies have shown that the therapeutic effects of MSC-EVs are equal to those of the MSCs. The use of EVs rather than MSCs protects against issues of tumorigenicity, immunogenicity, and poor engraftment. In addition, EV-based therapeutic manufacture, as well as transport and storage, promises to be more readily scalable at lower cost than live cell MSC therapeutics. However, to achieve their full clinical potential and to avoid some of the pitfalls that have contributed to clinical trial failures of MSC therapeutics, EVs need to be thoroughly investigated in terms of purification, quantification, characterization and potency before their clinical use. Genetic modification of the source cells for large scale manufacture of MSC-EVs may serve as an effective strategy to further improve the therapeutic effect of MSC derived cell-free products. The increasing sophistication with which EVs from a variety of cell sources can be engineered, to enhance their homing capabilities and bolster their therapeutic cargo, further demonstrates the considerable potential of this versatile therapeutic platform.

## Author Contributions

RK, JJ, JMC, and MS designed and wrote the content, and revised the manuscript. All authors contributed to the article and approved the submitted version.

## Conflict of Interest

MS, JJ, and RK are employed by Exopharm Ltd. JMC is a contractor to Exopharm Ltd.
